# Rapid Maxillary Expansion and Upper Airway Morphology: A Systematic Review on the Role of Cone Beam Computed Tomography

**DOI:** 10.1155/2017/5460429

**Published:** 2017-07-16

**Authors:** Gabriele Di Carlo, Matteo Saccucci, Gaetano Ierardo, Valeria Luzzi, Francesca Occasi, Anna Maria Zicari, Marzia Duse, Antonella Polimeni

**Affiliations:** ^1^Department of Oral and Maxillofacial Sciences, Sapienza University of Rome, Rome, Italy; ^2^Department of Pediatrics, Sapienza University of Rome, Rome, Italy

## Abstract

**Objective:**

This study aimed to investigate the quality of cone beam computed tomography (CBCT) studies evaluating the effects of rapid maxillary expansion on upper airway morphology.

**Materials and Methods:**

A database search was conducted using PubMed, Ovid, and Cochrane Library up to December 2016. Studies in which CBCT was adopted to visualize the upper airway before and after rapid maxillary expansion were included. The population target was growing patients. Methodological quality assessment was performed.

**Results:**

The screening process resulted in the exclusion of 1079 references, resulting in only 9 remaining papers that fulfilled the inclusion criteria. No randomized clinical trials were found. The quality scores ranged from 36% to 68% of the maximum achievable, and the mean quality score of the studies was 50%. No good quality studies were detected in our sample.

**Conclusions:**

Inconsistencies in the CBCT protocols utilized were detected between studies. Head posture, tongue position, and segmentation protocols were not consistent. These discrepancies were reflected in the different results obtained in the studies. A valid and consistent protocol with regard to head and tongue positioning, as well as nasal cavity volume segmentation, is required.

## 1. Introduction

In the field of orthodontics, the classical studies performed on airway and craniofacial morphology using plane radiography in the 1970s have received renewed interest after the introduction of cone beam computed tomography (CBCT) [1, 2]. Although magnetic resonance (MR) and computed tomography (CT) were available before its introduction, CBCT is now the most commonly used technology to acquire digital data on the anatomy of the nose and pharynx in dentistry. The reduced costs and radiation dose for the patient compared to magnetic resonance imaging (MRI) and CT have contributed to the increased use of this technology [3, 4].

In the field of orthodontics, CBCT has added value when upper airway definitions are required for diagnosis and treatment planning [5]. Moreover, the use of software reconstruction in three dimensions enabled the manipulation of images in a three-plane space [6, 7]. These features enhance studies aiming to assess upper airway volume and morphology with respect to craniofacial growth, as well as maxillofacial surgical and orthodontic interventions [3, 4].

In this context, the dentoskeletal effects of rapid maxillary expansion (RME) have been extensively studied using different methodologies, from manual measurement of dental casts to plane lateral cephalometrics [5, 6]. Nevertheless, the drawbacks that characterize plane radiography, in particular the superimposition aspects, hinder efforts to depict the three-dimensional effects of RME treatments with respect to the nasopharyngeal cavity.

The interest in maxillary expansion is mainly clinical. Maxillary constriction can play a role in the development of obstructive sleep apnea (OSA), and in childhood, it may often be related to the existence of allergic rhinitis [7–16]. Recently, RME has been advocated as a treatment modality for OSA. Some authors claim that the associated nasal cavity volume increase after maxillary expansion leads to anterior repositioning of the tongue, resulting in an increase in the oropharyngeal space [11, 12].

Awareness of the possibility of increasing upper airway dimensions in order to prevent or relieve OSA symptoms in both adults and children led to an increase in the number of studies evaluating the outcomes of RME in terms of nasal cavity dimensions and upper airway patency via CBCT [13–16]. A recent review on maxillofacial surgery's effects on the upper airway confirmed that the introduction of CBCT significantly increased the possibility of obtaining more accurate information regarding the airway, although the application of this technology is not characterized by repeatability between studies, and there is also a relative lack of follow-up regarding the assessment of resulting modifications [8].

Given the recent increase in CBCT studies and the clinical relevance of RME in orthodontics and sleep medicine, it seemed reasonable to analyze, in a systematic review, the quality of the published studies investigating the effects of RME evaluated using CBCT. The questions we aimed to answer were as follows: is the application of CBCT coherent and reliable between studies? Is RME able to generate a significant volumetric increase in nasal and/or pharyngeal airway dimensions? Are these modifications stable?

## 2. Materials and Methods

The method used to conduct this systematic review was based on the PRISMA guidelines (http://www.prisma-statement.org/) [17]. The analysis method and inclusion criteria were specified in advance and documented in a protocol in order to restrict the likelihood of post hoc selective bias.

### 2.1. Eligibility Criteria

Eligibility criteria relating to the population, intervention, comparison, outcome, and study design (PICOS) are presented in Table 1. No minimum length of follow-up was included in the eligibility criteria. The predictor variable was RME, and the primary outcome was linear transversal and volumetric changes in the nasopharyngeal airway as measured via CBCT.

### 2.2. Search Strategy

In order to identify relevant studies investigating the impact of RME on airway morphology, a computerized database search was conducted using the Medline database (PubMed), Ovid, and Cochrane Library. The search covered the period up to December 2016. The filters applied were “English” and “human studies.” The search strings used were devised with the help of an expert bibliographer and were (“cone beam” OR “cone-beam” OR “tc” OR “ct” OR “computed tomography”) AND (“airway” OR “upper airway” OR “pharynx” OR “nasopharynx” OR “oropharynx” OR “nasal cavity”) AND (“rapid maxillary expander” OR “RME” OR “rapid” OR “maxilla” OR “maxillary” OR “expansor”).

### 2.3. Study Selection

The full articles selected based on the abstracts were required to indicate the use of CBCT to measure airway volume before and after intervention and the inclusion of patients in the growing period. Studies investigating surgically assisted RME therapy, bone-borne RME, dental expansion, subjects with cleft and lip palate and other craniofacial deformities, syndromes, subjects affected by OSA, or other concomitant treatment during RME therapy were excluded. Studies performed using MRI and CT were excluded because they were not consistent with the objectives of this review.

From the database thus generated, all titles and abstracts not related to the topic were excluded, as were articles classified as Author's Opinion, Annals, and Case Reports. The potential eligibility of studies was determined via a detailed review of the selected abstracts to identify those that were compliant with all the inclusion and exclusion criteria. If the abstract contained insufficient information for a final decision, two authors (GDC and MS) jointly analyzed the full text after independent selection. In cases of discrepancy, a discussion among the entire review team (GI, VL, and FO) was implemented in a consensus meeting. The reference lists of the selected articles were manually examined for publications that may have been missed in the database searches.

### 2.4. Quality Assessment

A methodological quality grading was used to identify which of the selected studies would be most valuable. The final sample was evaluated on the basis of study design, study measurements, and statistical analyses (Table 2). The grading process used was an adapted version of one previously used in a recent systematic review by Gurani et al. [18]. According to van Vlijmen et al. [5], the mean quality of studies can be rated as <60% = poor quality; 60%–70% = moderate quality; or >70% = good quality. The methodological quality scores were calculated as percentages of the maximum achievable score (22 points) for each study.

## 3. Results

### 3.1. Database Search Results

**Figure 1:**
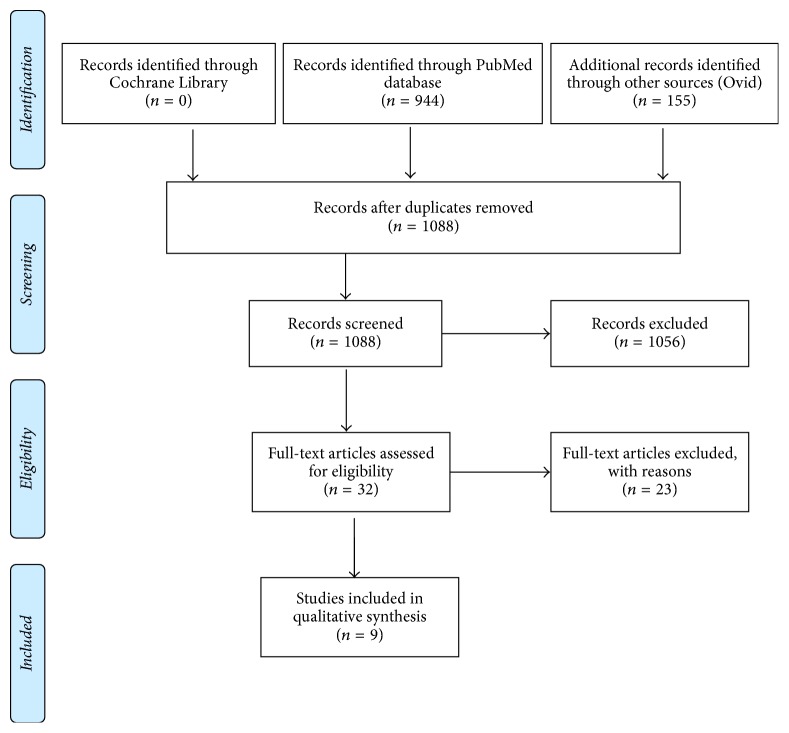
PRISMA 2009 flow diagram, from [17]. For more information, visit http://www.consort-statement.org.

A PRISMA flow diagram is shown in Figure 1. After duplicates were removed, there were 1088 references retrieved via the initial database search. Their titles and abstracts were screened. Particular attention was paid to the key study terms—rapid maxillary expansion/RME, cone beam computed tomography/CBCT, and nasopharyngeal airway reconstruction. The bibliographies of the included papers were reviewed. This did not result in any additions to the final list. The screening process resulted in the exclusion of 1079 references, leaving 9 full-text articles.

### 3.2. Study Characteristics

A brief summary of the 9 articles included is shown in a PICOS table (Table 3). The 9 articles were all published between 2010 and 2014. The samples sizes of the studies ranged from 14 to 35. Six studies were prospective, and three were retrospective. All 9 included significant variations in the methodology applied. The mean initial age of the experimental groups combined was 11.3 ± 2.1 years (range 7.5–12.9). The expander types used included the Haas expander [19, 20], the Hyrax expander [21–24], and the McNamara type expander [6]. Iwasaki et al. [25] did not specify the type of maxillary expander used, and Pangrazio-Kulbersh et al. [26] included both Hyrax and McNamara type expanders in their study. The CBCT devices used to acquire images were the I-CAT [6, 19, 20, 26], CB-Mercuray [25], Vatech [23], InVivo [24] Scanora [22], and Newtom [21]. The software packages used for three-dimensional reconstruction were Dolphin [6, 19, 20, 22, 24, 26], Intage [25], V-WORK [21], and EZ-3D [23]. The activation protocols and follow-up periods are shown in Table 4. In addition to a static analysis (linear, cross-sectional, and volumetric analysis), Iwasaki et al. [25] performed a fluid dynamic evaluation at the level of the nasal cavity.

### 3.3. Results of Quality Assessment

The methodological quality score results are shown in Table 2. None of the studies met all the requirements in our specific methodological assessment. None of the studies reported the randomization of their sample. Only Zhao et al. [21] adopted a blinding procedure when measurements were conducted. With regard to sample size, only El and Palomo [24] met the requirement of our methodological assessment. Only El and Palomo [24], Zhao et al. [21], Pangrazio-Kulbersh et al. [26], and da Baratieri et al. [20] included control groups. The methodological quality scores ranged from 36% to 68% of the maximum achievable score, and the mean quality score of the studies was 50%. No good quality studies were detected in our sample. Only Zhao et al. [21], El and Palomo [24], and da Baratieri et al. [20] could be classified in the range of “moderate quality” according to van Vlijmen et al. [5].

## 4. Discussion

### 4.1. CBCT Protocol

The present review aimed to investigate the existence of solid and coherent protocols when CBCT was adopted to measure airway dimensions and morphology in subjects undergoing RME. There was wide heterogeneity between the CBCT methodologies used in the studies. With regard to head position, the natural head position (NHP) is the suggested standardized position [18]. In our sample, NHP was adopted by Chang et al. [22], Zhao et al. [21], and Iwasaki et al. [25]. However, it has to be taken into account that, for repeatable measures of upper airway volumes, the NHP may be difficult to determine clinically. Given this, Zeng and Gao [23] utilized a cervical collar to control head position, in an effort to minimize systematic errors at the time of acquisition. The different methods used to ensure repeatability in terms of head position reflect a lack of valid information on how deviation from the NHP may influence upper airway dimensions during CBCT acquisition.

Tongue position is a relevant issue when assessing the airway using CBCT. There was a lack of information in this respect in all the studies included in the current review. Breathing and its influence during acquisition are quite difficult to control, particularly when dealing with children [24, 25]. The likelihood of achieving adequate control over tongue position, which may be affected by swallowing and breathing, is inversely proportional to the gradual reduction of scansion time as stated by Guijarro-Martínez and Swennen [27].

Different CBCT machines were used in the studies in our sample. Only Zhao et al. [21] adopted a supine acquisition methodology. Whether a supine position or an upright position is best for imaging the upper airway using CBCT remains a subject of debate.

Though the upright position is closer to the NHP and is recommended for baseline assessment of upper airway morphology, a supine position is closer to the sleeping position, where collapse of the airway is more likely to occur, even though it is known that during sleep patients present different muscular tone than they do when they are awake [28, 29]. The currently available data are not sufficient to support the use of a supine position or an upright seated position during acquisition. In studies in which sleep apnea patients are being investigated, the supine acquisition should be considered the preferred method to scan upper airway.

One of the advantages yielded by reconstruction software is the ability to visualize a three-dimensional object that represents the void space and characterizes the nasopharyngeal airway space. From the three-dimensional object, it is possible to calculate the volume and the minimal cross-section of the airway space. For this reason, a major issue when measuring the upper airway via CBCT is the thresholding. Of the studies included in the current review, only 2 performed segmentation of the nasal cavity [24, 25]. Chang et al. have stated that as the nasal cavity contains multiple connected cavities, performing such segmentation is difficult [22]. Moreover, in most of the studies in the current systematic review descriptions of the parameters used regarding threshold definition were lacking.

We believe that linear measurements at the nasal pharyngeal cavity level are not able to depict the entire three-dimensional morphology, and the positioning of landmarks on the curved lateral wall of the nasal cavity lacks repeatability. The application of thresholding can be automatic or manual. Different studies have shown that a manual threshold value has to be individually determined for each CBCT scan [3, 27, 30]. Though this is a time consuming approach, this method has been deemed the most reproducible.

A previous review on RME and the airway published in 2011 claimed that there was no norm for airway volumes depending on head position and breathing stage [31]. The current review shows that this issue has still not been addressed in the present literature. Recently, this topic was addressed by Gurani et al. [18], who consistently claimed that tongue position and head position were underestimated as confounding factors.

### 4.2. Main Findings

Table 4 highlights the wide heterogeneity between the types of expanders, activation protocols, and mean ages of the subjects between the studies. A common finding in our review was the use of different anatomic boundaries for the evaluation of the upper airway; thus, comparisons between studies in this respect were problematic. Moreover, drawing conclusions on the stability of the effects obtained was complicated due to the different follow-up times between studies, as indicated in Table 4. Clear statements on follow-up were not consistently reported in the studies included in the current review. Moreover, Iwasaki et al. [25] did not specify the interval between the expansion and the second acquisition.

In terms of study design, 6 studies reported the adoption of a prospective design. Moreover, only 4 of the 9 studies used a control group. El and Palomo [24] evaluated a control group matched for age, sex, and length of treatment wherein the subjects were involved in an orthodontic treatment without an expander. da Baratieri et al. [20] used a sex- and age-matched paired control group that did not receive any treatment and scanned the subjects twice but no specific reasons for this were reported. Iwasaki et al. [25] used a group matched for age and dentition who received orthodontic treatment, and Zhao et al. [21] used controls matched for age and sex who received orthodontic treatment other than RME. Interestingly, only 2 studies included blinded measurement methods [20, 21]. Pangrazio-Kulbersh et al. [26] did not use a control group, although they compared two different types of expanders. The inclusion of a control group is useful when long-term follow-up is required, to rule out the effects of growth at the nasopharyngeal level. The inclusion of a group who received full fixed appliances as their only orthodontic treatment could be a viable option when other possible reasons for scanning the patients in the control group twice are lacking.

When evaluating the effects of RME on the nasal cavity, the intervention time seems to have a pivotal role. The expansion effect seems to be more favorable when it is performed before the pubertal growth peak [29]. In the studies in the current review, growth stage assessment was only described in Christie et al. who used hand-wrist radiographs, and da Baratieri et al. [20] and Pangrazio-Kulbersh et al. [26], who only included subjects at a stage prior to the pubertal growth peak. The use of the Cervical Vertebrae Maturation Stage method instead of hand-wrist radiography remains a subject of debate [32]. Nevertheless, the lack of information regarding growth status was one of the parameters that contributed to the low to moderate quality of the evidence presented in the studies included in the current review. These drawbacks, in addition to the heterogeneity regarding the CBCT parameters used, precluded the possibility of conducting a meta-analysis. Therefore, the results obtained are presented herein in the form of a narrative synthesis.

Christie et al. found a significant increase in nasal width assessed via linear measurements immediately after the end of an expansion activation protocol [19]. El and Palomo [24] did not find a statistically significant increase at the oropharynx level, and on the contrary, nasal airway volume was significantly increased in the treatment group compared to the control group. Notably however, in that study nasal volume was measured partially, excluding the superior part of the nasal cavity. Surprisingly, an assessment of total volume was not performed despite the fact that the total nasal cavity was segmented [24].

da Baratieri et al. [20] did report an increase in all the linear measurements obtained except the inferior cross-sectional area of the nasal cavity compared to control group, at a 1-year follow-up time-point. One limitation of that study was the two-dimensional approach used despite the fact that three-dimensional datasets were available using a similar two-dimensional approach; Zeng and Gao [23] reported a decrease at the oropharynx level, although it was not statistically significant. Conversely, the nasal cavity increased significantly, although the absolute increase was very small. The nasal width measurements increased after expansion.

Chang et al. [22], in contrast to El and Palomo [24], reported an absence of any increase in volume or cross-sectional area at the oropharynx level when a CBCT scan was taken 4 months postexpansion. They only detected a statistically significant increase in cross-sectional area at the level of the posterior nasal spine to the basin.

In contrast to El and Palomo [24], Ribeiro et al. [6] reported an increase at the oropharyngeal airway which may imply that tongue repositioning had taken place. An increase in the transversal linear measurements of the lower third of the nasal cavity was detected 4 months after the end of the activation protocol. Those results are of limited relevance, however, as acknowledged by the authors, due to the absence of a standardized acquisition protocol in terms of tongue position, head inclination, breathing, and swallowing.

Iwasaki et al. analyzed groups of patients subdivided based on obstruction and nonobstruction at the nasal cavity level. Obstruction was confirmed via computational fluid dynamics. They found that improvement of nasal airway ventilation obtained via RME was associated with improved low tongue positioning [25]. The improvement in airway volumes could not resolve the presence of an obstruction at the nasal cavity level. No difference in oropharyngeal volume was detected between the two groups.

Zhao et al. [21] found no evidence to support the hypothesis that RME treatment increases the volume of the oropharyngeal airway despite the increased intermolar width after RME treatment. Pangrazio-Kulbersh et al. [26] compared 23 prospectively treated patients treated using bonded or banded expanders. They did report an increase at the maxillary sinus level, although no difference in the oropharyngeal airway was detected after treatment. The airway was measured between the posterior nasal spine as the superior border and the epiglottis as the lower limit.

## 5. Conclusions

The use of CBCT was inconsistent between studies. A standardized protocol is required in order to avoid systematic errors with regard to head and tongue position during acquisition. Moreover, segmentation of the nasal cavity was an issue seldom considered in the studies included in this review, which often utilized a two-dimensional approach despite the fact that three-dimensional datasets were available. Characterization of the nasal cavity and the overall volume calculation should be not overlooked when DICOM files are available. Randomized clinical trials incorporating blinded measurement approaches are needed, in order to establish the role of maxillary expansion with respect to nasopharyngeal airway morphology. Moreover, additional evidence of the stability of the effects of RME is required.

## Figures and Tables

**Table 1:** PICOS description.

Population	Clinical patient studies that evaluated the effects of specific rapid maxillary expansion on the volume of the nasopharyngeal airway
Intervention	Rapid maxillary expansion evaluated via cone beam computed tomography
Comparison	Age- and sex-matched subjects who did not undergo rapid maxillary expansion therapy
Outcome	Changes in the dimensions of the nasopharyngeal airway
Study design	Randomized and nonrandomized controlled trials and observational studies. Case reports and author's opinion publications were excluded

**Table 2:** Quality assessment.

PAPER	Christie et al., 2010	Zeng and Gao 2013	El and Palomo, 2014	Zhao et al., 2010	Iwasaki et al., 2013	Chang et al., 2013	Ribeiro et al., 2012	Pangrazio-Kulbersh et al., 2012	da Baratieri et al., 2014
*Study design*									
Time	2	2	1	1	1	1	2	2	2
Randomized sample	0	0	0	0	0	0	0	0	0
Control group	0	0	1	1	0	0	0	1	1
Sample size	0	0	1	0	0	0	0	0	0
Sample gender	0	1	1	0	0	0	0	0	2
Objective	1	1	1	1	1	1	0	1	1
Selection criteria	1	1	1	1	1	1	0	1	1
Baseline characteristics	0	0	0	1	0	1	0	0	1
*Study measurements*									
Segmentation method	0	0	1	1	1	1	0	0	0
Type of airway measurements	1	2	3	4	2	3	3	1	2
Blinding measurements method	0	0	0	1	0	0	0	0	1
*Data analysis*									
Statistical analysis	0	1	1	1	1	1	1	0	1
Validation of measurements (error of the method)	1	1	1	1	1	1	0	1	1
Data presentation	2	1	2	2	2	2	2	2	2

*Total points*	**8**	**10**	**14**	**15**	**10**	**12**	**8**	**9**	**15**
*% score*	**36**	**45**	**63**	**68**	**45**	**54**	**36**	**40**	**68**

**Table 3:** Summary of included studies.

Study	Participants	Interventions	Comparisons	Outcomes	Study design
Christie et al.	Twenty-four healthy children (mean age, 9.9 years; range, 7.8–12.8 years; 14 boys, 10 girls).	Haas-type maxillary expander. Expansion was carried out as 2 turns per day (0.2 mm per turn) until the required expansion was complete	None	From T1 to T2, mean increases in nasal width, significant increases in basal bone of the maxilla, and significant openings in the midpalatal sutures were found	Pretreatment orthodontic records (T1) and immediately after expansion (T2) CBCT images were taken for all patients

Zeng and Gao	16 children (10 male; 6 female) with a mean age of 12.73 1.73 years (range, 10–15 years)	All subjects used 4,6-banded hyrax expanders. The active expansion period ranged from 2 to 3 weeks according to the expansion amount (2.7–6.3 mm). The retention period lasted 3 months	None	Linear width measurements at the level of nasal cavity increased. Lower nasal volumes increased; on the other hand, oropharyngeal volumes decrease though without any significance	CBCT images were taken immediately before (T1) and three months after expansion (T2)

El and Palomo	Two groups were selected, each with 35 patients (15 males, 20 females): Rapid Maxillary expansion group and a control group.	Expansion protocol consisted of twice per day screw activation until a slight amount of overcorrection was achieved. The retention period lasted 4–6 months	The control groups were matched for age, sex, and treatment duration	A statistically significant airway value was seen in both groups although only nasopharyngeal airway showed significant difference between the groups	CBCT data for pretreatment (T0) and posttreatment (T1) intervals. Linear, cross-sectional, and volumes along airway passage were assessed

Zhao et al.	The experimental group consisted of 24 patients (mean age, 12.8 ± 1.88 years) with maxillary constriction who were treated with hyrax palatal expanders	Hyrax type palatal expanders turned 1 or 2 times per day until the required expansion was achieved, retention for at least 3 months after expansion	The control was paired in age and sex; patients included were just starting regular orthodontic treatment	Only retropalatal airway volume was found to be significantly different between groups before treatment, and this difference remained after treatment. No other statistically significant differences were found for the airways measurements	CBCT scans were taken of all patients as part of both initial orthodontic treatment records and progress records

Iwasaki et al.	Twenty-eight treatment subjects (mean age 9.96 6 1.21 years) who required RME treatment had cone-beam computed tomography images taken before and after RME.	Rapid maxillary expansion with 5 mm of expansion in average	The control group consisted of serial CBCT images of 20 subjects (8 boys, 12 girls) with no history of RME appliance treatment	Intraoral airway volume (tongue posture) decreased significantly in the RME. The increase of pharyngeal airway volume in the control group was only 41% that of the RME group	CBCT data were taken before and after RME treatment (RME group) or at corresponding times but without RME treatment (control group)

Chang et al.	Fourteen orthodontic patients (mean age, 12.9 years; range, 9.7–16 years) were recruited.	The activation protocol consisted of 1 activation (90 turn) of the jackscrew per day for 28 consecutive days or until resolution of the posterior crossbite	None	The cross-sectional airway measured from the posterior nasal spine to basin level was the only parameter showing a significant increase after rapid maxillary expansion	The initial CBCT scan was taken 0 to 14 days before cementation of the maxillary expander, and the progress CBCT scan was taken 3 to 4 months after completion of active maxillary expansion

Ribeiro et al.	15 mixed dentition individuals (8 females and 7 males)	All patients were treated with rapid maxillary expansion using a fixed appliance with occlusal acrylic coating	None	The nasal cavity presented a significant transversal increase at level of the lower third. No significant change occurred in the nasopharynx although they refer to retropalatal area. A significant change was noted in the oropharynx in volume immediately after the RME	Patients were evaluated before and 4 months after the RME

Pangrazio-Kulbersh et al.	23 prospectively treated patients. 13 subjects, 7 males and 6 females with a mean age of 12.6, were treated using banded expanders, and 10 subjects, 5 males and 5 females with a mean age of 13.5, were treated with bonded expanders	Each group had the same expansion activation protocol and retention time: expansion time was 4–6 weeks, with 6–10 mm of activation	The two expansion groups were compared	Both appliances equally increased the skeletal and soft tissue dimensions of the nasal cavity and maxillary sinus volume. The posterior airway volume did not significantly change with either method of expansion	CBCT images weretaken at T1 (pretreatment) and T2 at 6 months immediately after removal of the appliance

da Baratieri et al., 2014	It comprised 30 subjects (18 males and 12 females), mean ages 9 for males and 9.7 for females. Divided in expansion and control group	Rapid maxillary expansion using banded appliance	The activation group was compared with a paired age and sex, control group	All the linear (width) measures of nasal cavity increased significantly except for alveolar angulation and inferior nasal cavity area	CBCT taken at treatment onset and one year after expansion

**Table 4:** 

Studies	Mean age of intervention group	Type of expander	Activation protocol	2nd CBCT acquisition
Pangrazio-Kulbersh et al.	First group, 12.6; second group, 13.5	13 banded versus 10 bonded	4/6 weeks	After 6 months of retention
Riberio et al.	7.5 years	McNamara	NS	4 months after maxillary expansion
Chang et al.	12.9 years	Hyrax expander	1 activation for 28 days	3-4 months after maxillary expansion
Iwasaki et al.	9,96 years	NS	NS	1,27 years after first acquisition
Zhao et al.	12,8 years	Hyrax	1 or 2 times per day for 4/6 weeks	From 8 months to 2 years after first acquisition
El and Palomo	14 years	Hyrax	2-time activation per day	NS
Zeng and Gao	12,73 years	Hyrax	2/3 weeks	3 months after expansion
Christie et al.	9.9 years skeletal 10	Haas	30 days	Immediately after expansion
da Baratieri et al.	9.6 years	Haas	2-time activation per day	1 year after RME

NS, not specified.
